# A dynamically structured matrix population model for insect life histories observed under variable environmental conditions

**DOI:** 10.1038/s41598-022-15806-2

**Published:** 2022-07-08

**Authors:** Kamil Erguler, Jacob Mendel, Dušan Veljko Petrić, Mina Petrić, Mihaela Kavran, Murat Can Demirok, Filiz Gunay, Pantelis Georgiades, Bulent Alten, Jos Lelieveld

**Affiliations:** 1 The Cyprus Institute, Climate and Atmosphere Research Centre (CARE-C), 20 Konstantinou Kavafi Street, 2121 Aglantzia, Nicosia, Cyprus; 2 Department of Medical Sciences, University of Oxford, Oxford, UK; 3 Laboratory for Medical and Veterinary Entomology, Faculty of Agriculture, University of Novi Sad, 21000 Novi Sad, Serbia; 4 Avia-GIS NV, 2980 Zoersel, Belgium; 5 Biology Department, Ecology Division, VERG Laboratories, Faculty of Science, Hacettepe University, 06800 Beytepe-Ankara, Turkey; 6 Max Planck Institute for Chemistry, 55128 Mainz, Germany

**Keywords:** Climate-change ecology, Ecological modelling, Population dynamics, Theoretical ecology, Software

## Abstract

Various environmental drivers influence life processes of insect vectors that transmit human disease. Life histories observed under experimental conditions can reveal such complex links; however, designing informative experiments for insects is challenging. Furthermore, inferences obtained under controlled conditions often extrapolate poorly to field conditions. Here, we introduce a pseudo-stage-structured population dynamics model to describe insect development as a renewal process with variable rates. The model permits representing realistic life stage durations under constant and variable environmental conditions. Using the model, we demonstrate how random environmental variations result in fluctuating development rates and affect stage duration. We apply the model to infer environmental dependencies from the life history observations of two common disease vectors, the southern (*Culex quinquefasciatus*) and northern (*Culex pipiens*) house mosquito. We identify photoperiod, in addition to temperature, as pivotal in regulating larva stage duration, and find that carefully timed life history observations under semi-field conditions accurately predict insect development throughout the year. The approach we describe augments existing methods of life table design and analysis, and contributes to the development of large-scale climate- and environment-driven population dynamics models for important disease vectors.

## Introduction

Life tables provide valuable insights into the environmental dependence of many species^1^. They have been used to inform species conservation^2^ and vector and pest control^3,4^. By providing the biological foundations, they have become essential components of mathematical models that predict population dynamics and disease risk^5–8^.

A common practice in constructing life tables is to follow a cohort of organisms under controlled conditions and catalogue the number of individuals at each development stage together with a set of processes, such as mortality and fertility^1^. Several statistical methods have been derived to infer realistic development time distributions and survival rates from life history observations^9^. Some of the early pioneers assumed normal or gamma distributions for stage durations and a time-varying survival probability^9–11^.

For many insect vectors, including mosquitoes, ticks, and small biting flies, the assessment of viability during development can be challenging^12,13^. Population heterogeneity, as an added complexity, may arise from the overlap of subsequent development stages. Experimental interventions, *e.g.* separating individuals or development stages, to improve observations may interfere with the natural processes^14,15^. On the other hand, most insect species are strongly affected by external temperature variations as they lack the ability to regulate their body temperature^16^. Attempts to establish controlled experimental conditions are known to result in inferences which may not be applicable in the field under diurnal variability^17,18^.

Understanding the environmental dependencies of population dynamics thus presents an inverse problem, where observations inform mathematical models (as opposed to the forward problem of finding a solution to a model)^19^. Inverse problems involve mechanistic models, which represent a simplified version of the reality. To provide accurate insights into environment-driven population dynamics, models should ideally capture as much of the complexities of the biological processes, environmental drivers, and experimental methods^20^. Population heterogeneity, time delay of development, and variable environmental conditions could be accounted for in an ideal model. Finally, the computational demand of the mathematical framework should enable fast optimisation in a multi-dimensional parameter space^21,22^.

Several modelling frameworks have been employed for environment-driven population dynamics of insect vectors that transmit human disease. Some of the most popular ones are based on ordinary differential equations (ODEs)^6,23–26^. Despite their prevalence and relative ease of use, the canonical ODE framework poorly represents population heterogeneity and time-delay caused by development^27,28^. Extensions have been made to incorporate realistic development time distributions by incorporating a series of sub-stages with identical exponentially distributed dwell times (also known as the linear chain trick)^27,29,30^. Delay differential equations (DDEs) have also been used to model insect population dynamics with true time delays in development^31–33^. Although the framework implies a homogenous cohort, heterogeneity can be introduced at the beginning through variable entry times. Recently, the framework has been extended towards representing trait variation under environmental change in successive development stages and generations^34^.

Alternatively, discrete-time matrix population models (MPM) have been used to represent populations structured with respect to age^35^ or physiological stage^36^. MPMs make use of carefully designed projection matrices and matrix algebra to project population structure from one census date to the next^37,38^. Integral projection models (IPMs)^39^ and the age- and stage-structured model of Caswell et al.^40^ accommodate populations structured by a combination of age and a physiological trait, such as body size or wing length. Extensions have been made to accommodate a variable environment based on detailed age-trait observations^41–43^.

The age-structured population dynamics model of Erguler (sPop) is an MPM incorporating a dynamic age structure^44^. As specified by the model, individuals in a development stage are grouped into distinct age classes, and the population structure is propagated using hazard functions to generate the desired distributions of survival and development time. However, the effect of temperature on development is not exclusively age-dependent for many insect species, but it involves accomplishing a series of tasks through complex biochemical reactions, enabled by the heat absorbed^45–47^.

Here, we extend this framework by considering development as a cumulative process, where accumulation of a sufficient number of (intangible) units triggers stage completion. An analogy can be drawn to a mathematical concept called renewal processes^48^, where hypothetical events occur (arrive) randomly in time and iterate a process counter. By representing development as a renewal process, we extend the MPM framework with a dynamic state vector and projection matrix. As a result, the framework permits variations in both the number of pseudo-development stages and stage transition rates, and reproduce common development time distributions under the influence of environmental variation. To inform models based on this framework, we propose an experimental design of life table construction under variable semi-field conditions, which permits the inference of many environmental dependencies at once. We demonstrate this approach on two common disease vectors, the southern and northern house mosquito, *Culex quinquefasciatus* and *Culex pipiens*, respectively, and compare the information content of constant and variable-condition life table experiments.

## Methods

### sPop2: a dynamically-structured matrix population model

We represent development as a renewal processes^48^ with a dynamic probability of event arrival. While each renewal event corresponds to a fraction of development, individuals with identical development fractions are grouped together to form a pseudo-stage-structured population.

During a single time unit (time step), we assume that the arrival time of each event is a nonnegative independent identically distributed random variable. Therefore, the number of independent events arriving in a time step is a discrete random variable, *i*, with a cumulative density function $F(x,\theta ) = \Pr (i \le x|\theta )$, where $\theta$ represents the expected time between successive events (interarrival time). The fraction of development achieved with each event is 1/*k*, where *k* is the target number of events to complete development. Thus, the rate of accumulation—the fraction of development completed—per time step is given by *i*/*k*.

Under the specific restrictions of invariable *k* and deterministic dynamics, this framework is equivalent to an MPM. To expand from the matrix population framework, we introduced a development indicator, *q*, which is used to structure the population; *i.e.* individuals with identical *q* values are grouped together to form a pseudo-stage. A single indicator value of $q=0$ at the beginning of a simulation implies a cohort of individuals; however, a pre-structured initial population is also allowed. According to the framework, at each time step, *q* is incremented by *i*/*k*, and development occurs when $q \ge 1$.

We developed Algorithm 1 to facilitate the dynamic handling of the pseudo-stages, allow for intrinsic stochasticity, and accommodate variable interarrival times. We implemented the algorithm in C and distributed it under the GPL 3.0 license on the GitHub repository https://doi.org/10.5281/zenodo.5788377 as an extension to the age- and stage-structured population dynamics model of Erguler^44^ (sPop2 v.2.1).

Algorithm 1 simulates a single step of development in a deterministic or stochastic setting. The development scheme is selected by setting the cumulative density function, $F(x,\theta )$, according to (i) $I(x\ge \theta )$ for fixed duration, (ii) $1-\theta ^{x+1}$ for Pascal-distributed, and (iii) $\Gamma (x+1,1/\theta )/x!$ for Erlang-distributed development time. In this context, *I* is the indicator function and $\Gamma (x,\theta )$ is the upper incomplete gamma function. We present the detailed derivation of the three schemes in Development time distributions. To accomplish dynamic population restructuring in one pass, we employ the hazard function, $H(x,\theta )$, *i.e.* the probability of *x* events arriving conditional on the absence of fewer events,

1 $$\begin{aligned} H(x,\theta ) = \Pr (i = x \mid i > x - 1, \theta ) = \frac{F(x, \theta )-F(x - 1, \theta )}{1-F(x - 1, \theta )}, \end{aligned}$$

where

2 $$\begin{aligned} H(0,\theta ) = F(0, \theta ) \quad \text{and} \quad \lim _{x\rightarrow \infty }H(x,\theta ) = 1. \end{aligned}$$

Scheme selection is followed by iteration through the existing pseudo-stages. Here, we adopt the notation $\mathbf{q} = \left\{ q_0, q_1, \dots \right\}$ to represent the array of pseudo-stage classes and $\mathbf{n} = \left\{ n_0, n_1, \dots \right\}$ the individuals assigned to each class. At each step, each *q* is expanded into an array of pseudo-stage classes, $\mathbf{q'}$, as a result of incrementing *q* by *i*/*k* for every possible value of *i*. An appropriate fraction of the sub-population associated with *q* is allocated to each class in $\mathbf{q'}$ either deterministically or stochastically (given the probability of *i*). Once the smallest value of *i* such that $q'\ge 1$ is reached, the remaining individuals, *m*, are flagged to complete development, and the algorithm moves forward to the next *q*. As a result, for each *q* with an allocated population size of *n*, the algorithm generates one or more pseudo-stages ($\mathbf{q'}$) among which the population ($\mathbf{n'}$) is distributed accordingly.

It is important to note that the probability of *i* leading to $q>1$ in a time step may become significant especially with a relatively large arrival rate. Such overshooting will not prevent a population from completing development as required; however, may create bias should the process be followed by another stage of development. Overshooting can be minimised by extending the loop above $q=1$ to maintain a record of all individuals with $q>1$. In turn, the ones completing development form a structured population to be fed into the next stage of development. However, there may be cases where $q>2$ in one step, which require multiple successive stages to utilise the accumulated development. Consistent overshooting is a clear indication of a coarse time scale. However, due to the indirect time dependence of the renewal process, reducing the step size involves a change in the desired duration and rate of these processes (see Shifting the unit of time).
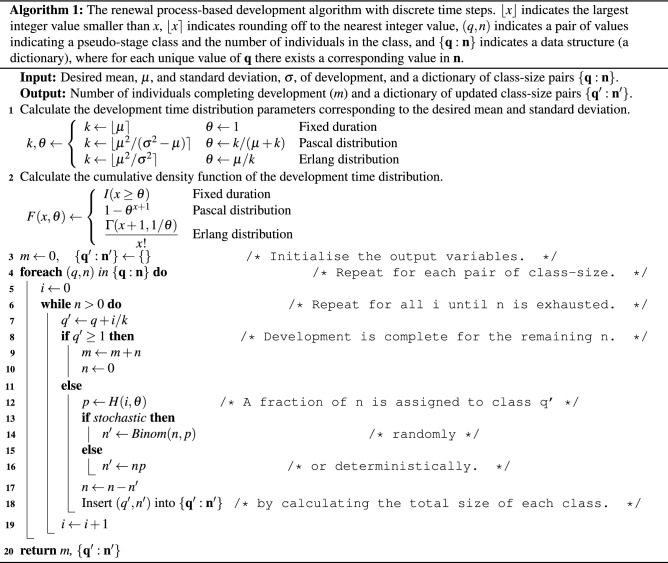


### Simulating development with renewal processes under variable conditions

We refer to development represented by renewal processes as cumulative development. To demonstrate how a change in rate manifests in cumulative development and how alternative representations capture the process, we constructed a scenario where a group of individuals is subjected to two different development rates. We employed a population of 100 individuals with a deterministic Erlang-distributed development time of $40\pm 5$ steps (a mean of 40 and a standard deviation of 5 steps corresponding to $k=64$ and $\theta =0.625$). At the $20^{th}$ step, we switched development time to $20\pm 5$ steps ($k=16$ and $\theta =1.25$) and continued simulation until all individuals complete the process.

We constructed four alternative models to compare with cumulative development. The first alternative is an age-structured MPM constructed using the sPop package^44^. The sPop algorithm dynamically structures a population with respect to the time spent in development, and the proportion of individuals completing development in a step ($d({\tau })$) is given by a gamma distribution with mean $\mu$ and standard deviation $\sigma$. We note that we use *t* to represent time in continuous domain and ${\tau }$ in discrete domain in this context. When we let $x_{\tau }$ represent the size of a population at step ${\tau }$,

3 $$\begin{aligned} \begin{array}{lcl} x_{{\tau }+1}= & {} (1 - d({\tau }))\,x_{\tau }, \end{array} \end{aligned}$$

where

4 $$\begin{aligned} \begin{array}{lcl} d({\tau }) &{} = &{} \left\{ \begin{array}{ll} {\frac{f({\tau }+1)-f({\tau })}{1-f({\tau })}} &{} \text{if}\,\,\, f({\tau }) \ne 1\\ 1 &{} \text{if}\,\, \,f({\tau }) = 1 \end{array} \right. , \end{array} \end{aligned}$$

$$f({\tau })=\gamma (k,{\tau }/\theta )/\Gamma (k)$$, $$k=\mu ^2/\sigma ^2$$, and $$\theta =\sigma ^2/\mu $$.

We employed the stage-structured DDE framework developed by Nisbet, Gurney, and Lawton as the second alternative^31,32^. By using the stagePop package written in R by Kettle and Nutter ^49^, we represented the process with a variable development duration function,

5 $$\begin{aligned} M(t) = \left\{ \begin{array}{ll} 1/60 &{} {\text{if}} \quad t<0 \\ 1/40 &{} {\text{else}} \end{array} \right. , \end{aligned}$$

and a rate of recruitment function,

6 $$\begin{aligned} R(t) = \left\{ \begin{array}{ll} f(t-20) &{} {\text{if}} \quad t<0 \\ 0 &{} {\text{else}} \end{array} \right. . \end{aligned}$$

We used *R*(*t*) to construct a population history with a gamma-distributed age-structure between $t=-40$ and $t=0$ to result in stage completion at $t=40\pm 5$. Since this is an artificial construct, we employed modified development rates for favourable (1/40) and unfavourable (1/60) conditions to yield approximately $20\pm 5$ and $40\pm 5$ units of development time, respectively. The R code implementing this model is given in The variable-rate development model in R..

The third alternative is an ordinary differential equation (ODE) of the form

7 $$\begin{aligned} \begin{array}{rcl} dx / dt= & {} -\lambda \,x, \end{array} \end{aligned}$$

where $\lambda$ is the reciprocal of average development time (shifting from 0.025 to 0.05 at $t=20$).

Finally, we introduced time dependence to the ODE by applying the linear chain trick (LCT)^27,29^, which results in

8 $$\begin{aligned} \begin{array}{rcl} dx_0 / dt &{}=&{} -\gamma \,x_0\\ &{}\vdots &{}\\ dx_k / dt &{}=&{} \gamma \,(x_{k-1}-x_k)\\ dx_{k+1} / dt &{}=&{} \gamma \,x_k, \end{array} \end{aligned}$$

where $$\gamma =1/\theta $$.

### Transformation of environmental variation in development

To investigate how frequent changes in the environment may affect development, and expand on the previous section, we assumed that there is a non-linear relationship between temperature and development rate. We employed the Briere-1 function, which is widely used to model development rate in insects^50^,

9 $$\begin{aligned} f_r(T) = \alpha \,T\,(T-T_L)\sqrt{T_H-T}, \end{aligned}$$

where $f_r(T)$ is the reciprocal of development time in days, *T* is temperature in degree Celsius, $T_L$ and $T_H$ are lower and higher temperature thresholds, and $\alpha$ is a scaling constant.

We conjectured a population of 100 individuals, with a deterministic Erlang-distributed (cumulative) development time, and defined the distribution with a variable mean ($\mu$) and a fixed standard deviation of 5 steps. We let $\mu$ be the reciprocal of $f_r(T)$, linking mean development time to temperature.

Next, we assumed that the population develops under a variable temperature regime, $\rho$, which is randomly sampled at each time step from one of the three alternative Gaussian distributions: (i) $\rho _L\sim N (15,4)$, (ii) $\rho _M\sim N (25,4)$, and (iii) $\rho _H\sim N (35,4)$, where $N (\mu ,\sigma )$ indicates a Gaussian distribution with mean $\mu$ and standard deviation $\sigma$, and $\sim$ defines the probability distribution of a random number.

### Population dynamics model of environment-driven insect development

We represented mosquito development with a generic 4-stage population dynamics model where eggs develop into larvae, then pupae, and emerge as adults. We assumed that egg, larva, and pupa development times are Erlang-distributed^9,10^, the mean of which are linked with ambient temperature using generic response functions, and assumed time-independent daily survival for each stage (see The population dynamics model in C for detailed implementation).

We linked mean development time ($\mu$) with temperature (*T*) with the widely used Briere-1 function (Eq. 9),

10 $$\begin{aligned} \mu = f_r(T)^{-1} = \left( \alpha _s\,T\,(T-T_0)\sqrt{T_1-T}\right) ^{-1}, \end{aligned}$$

where $T_0$ and $T_1$ are temperature thresholds ($T_1>T_0$), and $\alpha _s$ is a scale parameter. Since each dependency adds 3 parameters to the model, we assumed that the standard deviation of the distribution is proportional to its mean, *i.e.* $\sigma =\alpha _m\mu$, to limit the total number of parameters.

Furthermore, we employed a quartic (fourth degree) equation^7,51^ to represent the daily mortality rate, $p_m$, of each stage as a function of temperature,

11 $$\begin{aligned} p_m = p_0 + \alpha _s(T-Tm)^4, \end{aligned}$$

where $p_0$ is the lower boundary of mortality, $T_m$ is the optimum temperature, and $\alpha _s$ is a scale parameter. We clipped $p_m$ between 0 and 1 to represent a fraction. Overall, the generic model has 24 parameters, values of which will be inferred using life history data.

In addition to temperature, we assumed that development of insect larva may also depend on photoperiod^52–54^, and employed a generic sigmoidal relationship,

12 $$\begin{aligned} \begin{array}{lcl} \mu ' &{}=&{} \mu \times \phi \\ \phi &{}=&{} 1 + {\frac{\alpha _s}{1 + e^{\alpha _q(P-{\alpha _p})}}}, \end{array} \end{aligned}$$

where *P* represents day length at the corresponding latitude and date, $\alpha _p$ is the threshold, and $\alpha _s$ and $\alpha _q$ are scale parameters. We concluded this extended version with a total of 27 parameters. An illustrative C code implementing both the generic and extended models is given in The population dynamics model in C.

Further extensions to this model to represent the complete life cycle with egg laying is straightforward. Although we included an example model in the sPop2 v.2.1 package, we plan more in depth investigation of complete life cycles in future studies.

### Inverse modelling with approximate Bayesian computation

We followed the inverse modelling procedure described in Erguler et al.^55^, which involves proposing a generic model with exploratory environmental dependencies to be calibrated with observations using Bayesian principles. Accordingly, we propose a model, estimate an optimum parameter configuration based on an observational dataset, and sample a set of alternative configurations around the optimum using approximate Bayesian computation (ABC)^56^. To arrive at the optimum, we employed the hoppMCMC (v1.1) parameter optimisation and posterior sampling algorithm, which uses an adaptive basin-hopping Markov chain Monte Carlo (MCMC) method^57^.

We assumed a uniform prior, $\Pr (\theta )$, and replaced the likelihood function^58^, $\Pr (\delta |\theta )$, with a simulation-based distance (score) function, $f(\delta ,y(\theta ))$, where $\delta$ represents observations and $\theta$ is the parameter configuration of the model. A score function is a measure of similarity between $\delta$ and the model output, $y(\theta )$ (with parameter $\theta$). We employed the negative logarithm of the Poisson probability as the score function (SS),

13 $$\begin{aligned} SS = -\sum _t{\ln {Pois(\delta _t, \lambda =y_t(\theta ))}}, \end{aligned}$$

where $\delta _t$ is the observation at time *t* and $y_t(\theta )$ is the corresponding simulation using $\theta$. According to this, the posterior is approximated with a given threshold $\varepsilon$,

14 $$\begin{aligned} \Pr (\theta |\delta ) \approx \Pr (\theta |SS\le \varepsilon ), \end{aligned}$$

and it tends to its true value as $\varepsilon \rightarrow 0$. Despite the use of approximation, exploring the entire posterior distribution is computationally demanding, particularly when the number of parameters is large. To reduce the computational demand, we generated partial posterior samples around the optimum, *i.e.* sampled from the posterior mode, $\Theta$^8,55,59^.

### Life table experiments under constant conditions

In 2009 and 2010, Gunay et al. reported *Cx. quinquefasciatus* (Say, 1823) life tables constructed under constant laboratory conditions at five temperatures (15, 20, 23, 27, and 30 °C)^60,61^. To calibrate the generic model against these experiments, we employed the parameter optimisation/posterior mode sampling procedure with three replicates from each temperature regime. Following the experimental protocol, we initiated cohorts with 750 larvae and simulated development until adult emergence. As a result, we obtained 100 parameter samples from a posterior mode we labeled as $\Theta _q$ at $\varepsilon =2500$.

### Semi-field life table experiments

To evaluate development under variable conditions, we designed life table experiments for *Cx. pipiens* biotype *molestus* under variable semi-field conditions at the premises of the University of Novi Sad in Petrovaradin, Serbia. We introduced 16-to-24 h old egg rafts of the local laboratory colony in three Bioquip© mosquito breeders, set and kept outside in the semiurban (house garden) environment, from 1 February to 31 December 2017. We recorded hatching, transition of instars through the larval stages (L1 to L4), pupation, and adult eclosion time every day at 8 am local time. We added a pinch of Tetramine™ fish food after observing larvae hatching from the eggs. We obtained five-minute temperature readings of the ambient air from the weather station close to the breeders (1 m). New egg rafts were introduced after all larvae/pupae died or adults emerged, and a total of 16 experiments were performed (Fig. S4). We note that the initial conditions of the experiment shown in Fig. S4(b) were missing, and we estimated the number of eggs with a Poisson probability, assuming $30\%$ mortality according to Spanoudis et al.^62^. We followed the inverse modelling procedure and used seven of these experiments—one from (a), (b), (d), one from (f), one from (g), (i), and (j) shown in Fig. S4—to obtain 100 parameter samples from the optimum posterior mode, $\Theta _p$ ($\varepsilon =1600$), for the extended population dynamics model.

### Development profiles for *Cx. pipiens*

We define a development profile as a summary of development dynamics under variable conditions: maximum adult production, adult emergence time (development time), and the time when the first adults emerge in a series of experiments spanning a calendar year. We define development time as the time when half of the maximum adult production is complete. While adult production and development time pertain to each semi-field experiment, first emergence is defined for the calendar year.

To obtain a profile for *Cx. pipiens*, we simulated the development of 100 eggs (with the extended model and the samples from $\Theta _p$) until all the immature stages emerge into adults or die during the process. To extrapolate the profiles over a calendar year (annual development profile), we obtained hourly temperatures in Petrovaradin (45.2461012 latitude and 19.8516968 longitude) during 2017 from the ERA5 meteorological reanalysis dataset of temperature ($0.25^o$ spatial resolution)^63^. We converted the dataset into quarter-daily temporal resolution by averaging over every 6 hours, and simulated the development profiles at the beginning of each week.

## Results and discussion

### Renewal processes represent development under variable conditions

The consequence of a drastic environmental change can be demonstrated by introducing a shift in development time during the process. For demonstration, we consider a scenario where a group of individuals enter into a favourable environment reducing development time from $40\pm 5$ time units to $20\pm 5$.

**Figure 1:**
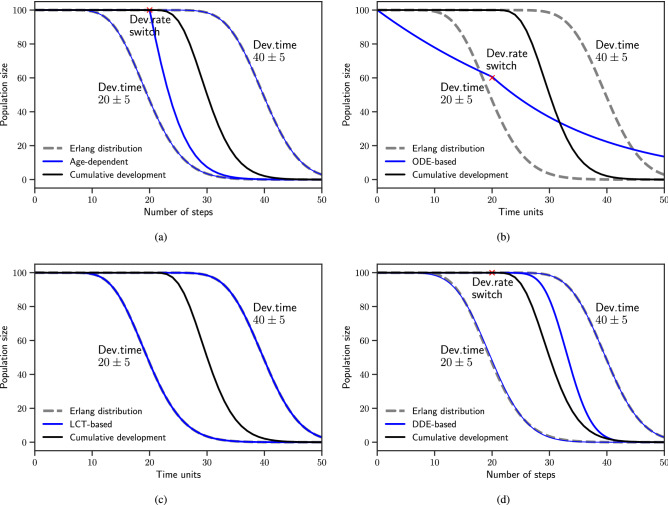
Response to change in development time. The number of developing individuals is simulated by using the cumulative development process and compared to (**a**) the age-dependent development process, (**b**) an ODE representation, (**c**) an LCT representation, and (**d**) a DDE representation. Solid dark lines show the cumulative development and thick blue lines show the alternative models. Dashed gray lines mark the two target trajectories before and after the shift in development time (marked with red crosses).

We show, in Fig. 1, that our dynamic pseudo-stage-structured MPM yields a gradual stage completion with an average development time of approximately $30\pm 5$ steps (solid dark lines) when conditions shift at ${\tau }=20$ (each step corresponds to 1 time unit). The target Erlang-distributed development trajectories without the shift are shown as dashed gray lines. The snapshots of the population structure, represented by the development indicator *q*, taken at each time step, show that half of the development is complete at the time of the switch and the switch accelerates the accumulation of *q* (Fig. S1).

In age-dependent development, a sharp transition, instead of a gradual one, is observed at the $20^{th}$ step (Fig. 1a). The switch results in the majority of individuals reaching target development age immediately at the time of switch. Previous work, reported in Erguler et al.^59^ and Erguler et al.^55^, aimed at modelling population dynamics under variable conditions, based on this dynamic age-dependent framework. Our results suggest that cumulative development might improve the fit to the data, prediction accuracy, and applicable geospatial range of these models.

We see in Fig. 1b that the canonical ODE framework represents an exponentially distributed development time and a shift in rate at $t=20$. The LCT extension to the framework helps to incorporate time dependence and represent the long and short development time distributions (Fig. 1c). The resulting model accommodates change in the rate parameter $\gamma$ (Eq. 8), *e.g* doubling of $\gamma$ changes development time from $40\pm 5$ to $20\pm 2.5$. However, to accommodate the required shift, the model needs to be transformed from a 66-dimensional system to an 18-dimensional one, which is beyond the scope of this work. We argue that in cases where development time distribution is fixed *a priori* (excluded from model calibration), the LCT framework provides a significant advantage over canonical ODEs. Although the framework has been used in the field of infectious disease epidemiology^64,65^, it has recently been applied to the modelling of vector population dynamics^30^.

The DDE framework also yields a gradual development trajectory with an intermediate duration (Fig. 1d). However, the distribution tends towards the longer development trajectory compared to the one achieved with cumulative development. The canonical DDE framework assumes a homogenous cohort, where all individuals react in the same way to variations in development rate. The assumption gives rise to sharp stage transitions within a single generation if all individuals are introduced at the same time. As a potential workaround, it has been proposed to generate a plausible population history, through variable entry times, until the required (or observed) developmental variation builds up^31,32^. Variation in development rates then acts upon the population and results in the modification of the existing age-structure. It is worthwhile to mention that a recent extension to the DDE framework to accommodate trait variation in population dynamics^34^ might also accommodate changing development rates within a single stage; however, it has not yet been employed at this scale.

Cumulative development is in agreement with the widely known degree-day (DD) framework, where development time is predicted by the heat accumulating in organisms^46^. Although the rate of accumulation in response to environmental conditions varies considerably, the DD framework implies that the combination of two different rates yields an average development time (also seen with cumulative development in Fig. 1). Experimental evaluation of this will be the topic of future research.

It is worth mentioning that our dynamically structured renewal process-based MPM follows the assumption of random population heterogeneity^9,11^; namely, at the individual level, the future behaviour of an organism is not affected by its historical behaviour. However, trait variation within a population is prevalent in many species, and is known to impact population dynamics and species interactions^34,66,67^. Future development of our framework will consider improving upon this limitation.

### Environmental variation transformed into development times

**Figure 2:**
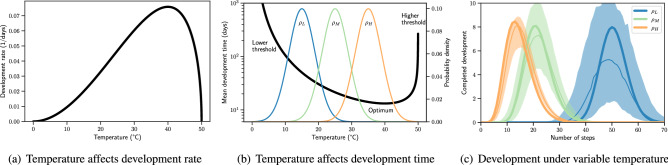
Development under environmental variation. In (**a**), development rate (Eq. 9) is shown with $\alpha =1.5\times 10^{-5}$, $T_L=0^oC$, and $T_H=50^oC$. In (**b**), mean development time is shown together with the probability densities of three temperature regimes ($\rho _L$, $\rho _M$, and $\rho _H$). In (**c**), the number of individuals completing development at each step are shown with respect to the three temperature regimes. Solid lines indicate the median, shaded areas indicate the $90\%$ range of 1000 simulations, and thick lines indicate simulations with the expected values of each regime.

Several non-linear relationships have been proposed to represent the temperature dependence of insect development^68^. A common feature is the presence of low and high temperature thresholds beyond which development is prohibitively slow. Often, there exists an optimum between the thresholds where the process is most efficient. A typical relationship between temperature and development rate, reported in Briere et al.^50^, is seen in Fig. 2a. Mean development time, given by the reciprocal of rate in Fig. 2b, exhibits the two thresholds and the optimum.

To investigate how temperature variation is transformed into cumulative development time, we assumed three variation regimes at relatively low, medium, and high temperatures ($\rho _L$, $\rho _M$, and $\rho _H$, respectively). Densities of the corresponding Gaussian probability distributions are plotted in Fig. 2b. Accordingly, each variation is transformed by a slightly different region of the rate function (Eq. 9). Eventually, the three development time distributions emerge as shown in Fig. 2c.

We found that the output of $\rho _H$ is skewed towards longer durations compared to what we would otherwise obtain if we simulated the process under constant conditions with the mean of $\rho _H$. The impact of variation in the middle range, $\rho _M$, is similar to that of $\rho _H$, but less pronounced. Conversely, the output of $\rho _L$ is skewed towards shorter durations. Our results suggest that, when development is already highly efficient, variation in temperature causes frequent encounters of longer (but not shorter) development durations, eventually extending the overall duration of the process. In the low efficiency range, development takes long to complete, but frequent encounters of relatively short durations—especially as the process approaches its optimum duration—triggers completion earlier than in the case of no variation.

Overall, our model predictions are in agreement with the rate summation effect, which states that the different outcomes obtained under constant and varying temperatures is due to the non-linear relationship between temperature and development rate (the Kaufmann effect)^16^. Furthermore, acceleration of development in insects subjected to varying high temperatures, its retardation at varying low temperatures, and low variability of development time in the linear range of the rate curve have been widely discussed^69^. Several groups have reported evidence in support of this effect, which is also in agreement with our results. For instance, Vangansbeke et al. (2015) reported for three insect species, *Phytoseiulus persimilis*, *Neoseiulus californicus*, and *Tetranychus urticae*, that varying temperatures with a lower mean yields faster development compared to the yield at mean constant temperatures^70^. However, observations of this phenomenon might result in different responses for different species at similar temperatures due to the difference in rate curves. Identification of the optimum temperature range may facilitate comparison. For instance, Carrington et al. (2013) assumed $26^oC$ as optimum based on the high dengue incidence in Thailand, and showed that large variations around $26^oC$ increases development time for the dengue vector, *Aedes aegypti*^71^. Wu et al. (2015) demonstrated that development is faster at around $26^oC$ compared to $23^oC$ for the fly, *Megaselia scalaris*, and found that varying temperatures at around $23^oC$ accelerates the process^47^. Finally, in a modelling study employing DDs, Chen et al. (2013) reported that larger diurnal temperature ranges relate to additional DD accumulation and faster development in grape berry moth, *Paralobesia viteana*^72^. Under the realistic non-optimum field conditions, where these simulations had been performed, a decrease in development time is expected in response to varying temperatures according to our results.

We note that the variation in development times is due to temperature since we ignore intrinsic stochasticity to demonstrate the impact of $\rho$ in isolation. The deterministic setup removes the upper limit in the number of distinct pseudo-stage indicators: a different *q* emerges from each *k*, and a different *k* emerges from each $\rho$. Since the number of pseudo-stages quickly exhausts the computational resources, we set the precision of *q* to the nearest 100$^{th}$ decimal point, effectively capping the number of pseudo-stages at 100 (see Accuracy of the pseudo-stage approximation). As shown in Fig. S2, the approximation has a negligible impact on accuracy.

### Environmental dependency extracted from life tables under constant conditions

Having discussed the importance of environmental variability in development, in this section, we employ a well-established experimental method to unravel the relationship between temperature and development time in a common mosquito species. In contrast to invasive vectors, which effectively render new territories suitable for disease transmission, *Culex* species pose an imminent threat with their wide distribution and ornitophilic (*Cx. pipiens* biotype *pipiens*), mamophilic (*Cx. pipiens* biotype *molestus*), and intermixed (their hybrids) blood feeding behaviour. Here, we investigate the temperature dependencies of mortality and development of *Cx. quinquefasciatus*, the southern house mosquito, which is an important disease vector, widely distributed across the tropics and sub-tropics^73,74^.

**Figure 3:**
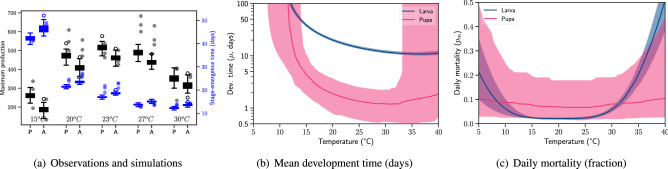
Inverse modelling of *Cx. quinquefasciatus* environmental dependency. The comparison of observed and simulated maximum pupa (P) and adult (A) production and the corresponding stage-emergence times is given in (**a**). Observations are represented with dots and simulations with box plots. The environmental dependency of larva and pupa development time (**b**) and mortality (**c**), derived by the posterior mode sample $\Theta _q$, is shown in (**b**,**c**). Solid lines represent the median and shaded areas represent the $90\%$ range.

To infer the dependencies, we used a generic temperature-driven insect development model, described in Methods, and the life history observations performed at five constant temperatures (15, 20, 23, 27, and $30\,^{\circ }$C) under laboratory conditions^60,61^. As a result of the inverse modelling procedure, detailed in Methods, we found that the generic model yields an overall match between the simulations and observations. In Fig. 3a, we present a comparison of observed and simulated maximum production and the stage-emergence times for pupae and adults. Here, we define the stage-emergence time as the time taken from the beginning of an experiment to the time when half of the maximum production of a stage (pupa or adult) is observed. In addition, in Fig. S3, we present the comparison of time trajectories separately for each temperature.

We found that the generic model faithfully replicates the observed development times of larvae and pupae. On the other hand, stage mortalities are predicted well at three temperatures, but are overestimated at 20 or $27\,^{\circ }$C. The impact of temperature on mortality might be more complex than it is captured by the quartic equation (Eq. 11). Optimum survival seen at $27\,^{\circ }$C suggests that the relationship might be non-symmetrical or multimodal. In addition, the observed variability in mortality suggests that the mismatch could also be due to experimental error or the intrinsic stochasticity of the biological processes.

We extracted the functional forms of temperature dependence from the posterior samples, shown in Fig. 3b, c, and found that the data inform the model as expected within the temperature range of the experiments ($15{-}30\,^{\circ }$C). Stage durations are well informed, and reflect the low variability seen in the data (the standard deviation is less than 1.5 days at all temperatures for both stages). Accordingly, pupae develop in less than 4 days, which is much shorter than the larva development time (between 10 and 20 days above $20\,^{\circ }$C). The model predicts that the minimum temperature at which development occurs (from the larva stage) is $10.5\,^{\circ }$C, which is close to $10.9\,^{\circ }$C, reported in Grech et al.^75^.

The observed variability in pupa and adult production suggests that survival is a highly stochastic process regardless of the controlled laboratory conditions. A deterministic model, such as the one used in this context, represents the mean of such processes but does not capture their variability. The simulated variability is a result of the uncertainty in parameter estimates. Model parameters contribute unequally to the output as a result of the model structure and the functional forms of temperature dependence, and the data inform certain parameters better than others^76,77^. For instance, daily mortality, shown in Fig. 3c, is more constrained for larva than pupa, which is likely due to the short duration of the pupa stage—changes in daily mortality have larger consequences as development time increases.

We note that a well-informed model yields predictions in the form of verifiable hypotheses; however, these are not necessarily accurate predictions. Model accuracy is assessed when such hypotheses are experimentally tested as part of the cyclic process of model development^78^. Here, we demonstrated that our modelling framework can be used to derive biologically meaningful inferences and to help improve the understanding of the temperature dependence of *Cx. quinquefasciatus*.

### Greater information content of semi-field experiments

The number of experiments required to test a range of conditions, including different combinations of multiple drivers, may quickly exhaust available resources. Moreover, variable conditions may have a previously unaccounted impact on development and mortality. In this section, we demonstrate that observations performed under variable conditions are valuable sources of information for our modelling framework, which is capable of representing the dynamics under such conditions.

*Cx. pipiens*, the northern house mosquito, is a competent disease vector, widely distributed across the temperate countries in North America, Europe, Asia, and North and East Africa^74,79^. Unlike *Cx. quinquefasciatus*, *Cx. pipiens* biotype *pipiens* is known to enter a reproductive diapause phase, where adult females arrest oogenesis during harsh winter conditions^80,81^. When larvae are exposed to short photoperiods and low temperatures during development, they emerge as adults destined to diapause. Although *Cx. pipiens* biotype *molestus* has lost the ability to diapause, its immature stages have been reported to retain metabolic sensitivity to photoperiod^82,83^.

To reveal the environmental dependence of the *molestus* biotype, we exposed its eggs to variable temperatures in semi-field conditions until adult emergence (or loss of cohort). The numbers of viable larvae, pupae, and adults observed in different experimental batches are given in Fig. S4. We employed the extended model with both temperature and photoperiod dependence (see Methods), and calibrated the model against seven of the semi-field experiments, performed in March, May, June, July, August, and September (Fig. S4(a), (b), (d), (f), (g), (i) and (j)).

As a result, we found that the model replicates the patterns of abundance emerging in the observations, *e.g.* stage timing and maximum adult production, reasonably well in most of the experiments, regardless of the times during which they were performed (Figs. S5 and S6). Quantitative evaluation of the agreement reveals that the observed and simulated adult emergence times are less than a week apart (Table 1).

**Table 1:** Comparison of observed and simulated adult emergence time and the total number of adults produced. Simulation output is given in terms of the median and $90\%$ range.

		Adult emergence			Adult production		
Id	Date	Simulation	Observation	Difference	Simulation	Observation	Difference
E1**	2017-03-16	–	–	–	0.00 (0.00–0.00)	–	–
E2	2017-03-23	–	–	–	0.00 (0.00–0.00)	–	–
E3**	2017-05-09	23.50 (22.00–24.52)	26.00	2.50	9.76 (6.51–13.84)	5.00	− 4.76
E4	2017-06-12	15.00 (14.25–15.75)	13.00	− 2.00	29.21 (26.65–32.26)	39.00	9.79
E5	2017-06-12	15.00 (14.25–15.75)	14.00	− 1.00	18.53 (16.90–20.46)	9.00	− 9.53
E6**	2017-06-12	15.00 (14.25–15.75)	15.00*	0.00	32.06 (29.25–35.40)	37.00	4.94
E7	2017-07-07	14.50 (13.50–15.75)	–	–	19.16 (13.12–22.56)	–	–
E8**	2017-07-11	15.25 (14.24–16.25)	19.00	3.75	7.17 (5.95–8.60)	10.00	2.83
E9	2017-07-11	15.25 (14.24–16.25)	16.00*	0.75	13.89 (11.52–16.65)	4.00	− 9.89
E10	2017-07-11	15.25 (14.24–16.25)	18.00	2.75	5.82 (4.83–6.98)	6.00*	0.18
E11	2017-08-04	14.25 (13.00–15.50)	13.00*	− 1.25	8.10 (4.67–10.67)	19.00	10.90
E12	2017-08-04	14.25 (13.00–15.50)	11.00	− 3.25	10.04 (5.80–13.24)	2.00	− 8.04
E13**	2017-08-06	16.00 (13.74–17.25)	13.00	− 3.00	25.11 (18.18–31.03)	17.00	− 8.11
E14**	2017-08-25	39.25 (32.25–41.60)	45.00	5.75	5.86 (4.55–7.23)	11.00	5.14
E15	2017-08-28	46.25 (44.20–49.25)	41.00	− 5.25	2.86 (2.03–3.79)	3.00*	0.14
E16**	2017-08-28	46.25 (44.20–49.25)	42.00	− 4.25	5.96 (4.24–7.90)	6.00*	0.04

*Observation within range

**Used for model calibration.

On the other hand, we found that egg and larva mortalities, and also, pupa and adult production are highly variable in the observations (see Fig. S4(c), (f), and (g)). Spikes of larva mortality are seen in Spring and Autumn (especially in May, September, and October). Despite this variability, the difference between the predicted and observed adult production was around 11 or less, except in the case of the experiment E7, which unexpectedly yielded only one pupa and no adults.

We obtain relatively large mismatches when predicting larva abundances, specifically where egg mortality is not predicted well (E5, E7, E8, E10, E11, E12). We hypothesise that the stress associated with rearing lab-grown specimens under variable conditions might elevate egg mortality, induce premature hatching, or affect the survival of the larvae produced. Since egg development starts inside gravid females, *i.e.* under the optimum conditions of the laboratory, the observable part of development subjected to variable conditions remains mainly the hatching behaviour. Consequently, we observed rapid and synchronous completion of the egg stage in all experiments (see Figs. S5 and S6). Being exposed to a narrow range of temperatures, relatively less information can be obtained on the environmental dependency of the egg stage. As a potential improvement, we recommend that future adaptations of the semi-field experiments consider using field-captured adult female mosquitoes as the source of eggs.

In addition to egg mortality, we observed spikes of larva mortality in May (E3), July (E8), and in Autumn (E14, E15, and E16). A likely cause of such transient high mortality is brief temperature shifts towards the extremes. However, the rarity of such events prevents the inverse modelling procedure from adequately capturing their impacts on life processes. As a potential improvement, we recommend that the experiments are performed in overlapping time frames, increasing the likelihood of observing the impact of an extreme event at different times during development. We note that the early decline in larva abundance seen in Autumn could be a result of insufficient food supply due to the increased nutritional requirements. According to the proposed metabolic response to short photoperiods, larvae would require additional food to accumulate fat reserves in preparation for diapause, the state where adult females endure several months without feeding. This implies that development takes longer than it would at long photoperiods when subjected to similar temperature regimes.

**Figure 4:**
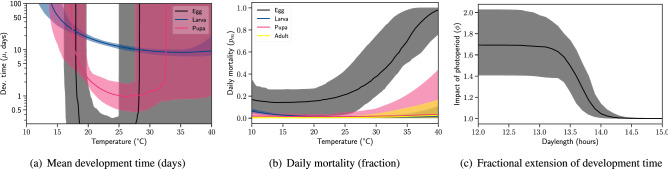
Environmental dependency of *Cx. pipiens* development and mortality inferred from semi-field life table experiments. Solid lines represent the median and shaded areas represent the $90\%$ range.

Using the extended model and the semi-field data, we identified the environmental dependencies shown in Fig. 4. The data informed about the temperature dependency of each life stage as well as the photoperiod dependency of larvae. As expected, the overall variability in the inferred dependencies is higher for *Cx. pipiens* compared to *Cx. quinquefasciatus* (Fig. 3). We found that the larva and pupa development times closely match the observations reported by Spanoudis et al.^62^ at long photoperiods (see Fig. S7). However, the development times reported in Kiarie-Makara et al.^84^ at short photoperiods and moderate temperatures do not suggest a significant impact of daylight, which could be due to the particular strain of *Cx. pipiens* used in these experiments. As expected, the temperature dependency of egg development was not well informed by the data in the current configuration of the model and the functional forms of environmental dependence.

We found that the photoperiod dependency is significantly non-linear with an average threshold of 13.7 hours of daylight (Fig. 4c). Photoperiod-driven extension in development time (about 1.7 times more at 13:11 h L:D than at 15:9 h L:D) contributes to improving the accuracy of predictions at the end of the high season (Fig. S8). The critical photoperiod (CPP) agrees well with the ones identified for *Cx. pipiens* biotype *pipiens*^85,86^. For instance, Sanburg and Larsen reported that there is an exponential relationship between follicle sizes in adult females (signifying commitment to diapause) and the photoperiods they were exposed to during immature stages^85^. We inferred a similar (but reverse) gradient between photoperiod and the extension of larva development time from 15 to 12 hours of daylight (Fig. 4c).

### Risk assessment with annual development profiles

**Figure 5:**
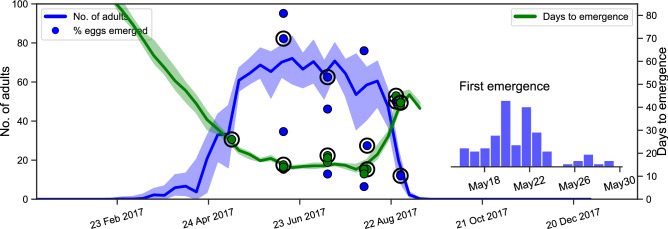
Annual development profile of *Cx. pipiens* in Petrovaradin, Serbia, in 2017. The outcome of each hypothetical semi-field experiment is plotted vertically along the y-axis at the date when the experiment is initiated. The maximum number of adults produced is given in blue, and the time it takes (from the date indicated on the x-axis) to produce half of the maximum is given in green. Solid lines represent the median and shaded areas represent the 90% range of model predictions. Outcomes of the semi-field experiments (dots) are plotted together with the model predictions. The time points marked with circles indicate the experiments used to calibrate the model. Estimated time of first adult emergence is given in the inset.

We extrapolated the development dynamics of *Cx. pipiens* over the calendar year by setting up a hypothetical experiment at the beginning of each week. We simulated the subsequent development dynamics and obtained the annual development profile as shown in Fig. 5. Accordingly, the immature stages begin development from late February and the first adults emerge in May (adults emerging late in May start developing in the experiments set up late in March). The profile is consistent with the regular *Cx. pipiens* high season in the region.

As seen in Fig. 5, predicted adult emergence times agree well with the observations throughout the high season. However, there is a greater variation in the maximum number of adults than the times of emergence (extending to almost $40\%$ of the possible outcomes in early August). A greater variability (almost $80\%$ in August) is seen in the corresponding observations, which we transformed into the percentage of eggs emerging as adults (where available) to facilitate comparison. According to the model, variation in adult production is associated with the variation in both development times and mortality during immature stages. We recall that the uncertainty in the informed environmental dependencies is high around relatively less frequently encountered values—especially the lower and higher temperature extremes (Fig. 4). Specifically, egg development times cannot be identified precisely, but immediate hatching of the larvae is predicted between 20 and 25 °C. Consequently, we found that frequent exposure to temperatures outside the well-informed range have a significant impact on the variation in adult production (Fig. S9).

We adopt the time of first adult emergence as a proxy of the first generation of adults in the season. According to our model, early adult emergence is a result of shorter development times and higher success rates, which indicates that the temperature conditions allow for an early first generation of adults. An early first generation greatly contributes to an early peak of adult abundance, which may increase the risk of vector-borne disease transmission in humans. For instance, an early peak of abundance may cause an early start of West Nile virus circulation and amplification in *Culex pipiens* and their avian hosts, which increases the likelihood of virus spillover to humans^51,87^. Anecdotal evidence shows that the anomalously hot April and May that occurred in 2018 in Serbia shifted the peak of *Cx. pipiens* abundance forward by more than one month (Petrić et al., unpublished). Similarly, 2018 was the year with the largest number of autochthonous West Nile virus infections throughout Europe (more than the total of the previous seven years together)^88,89^.

In summary, our results showed that the semi-field experiments, when used in combination with our dynamic pseudo-stage-structured MPM, help to develop predictive models and inform over a wide range of environmental conditions. We developed a predictive model of *Cx. pipiens* biotype *molestus* development and gained insights into the specifics of temperature and photoperiod dependencies by reducing the need of extensive laboratory data. We used life history observations from 7 experiments performed under semi-field conditions and employed a generic model structure, largely uninformed on the specific environmental dependencies of the species. The cumulative development framework we introduced applies broadly to poikilotherms subjected to highly variable environmental conditions. Although the generic model structure helps to develop exploratory models and identify potential environmental dependencies, accuracy can be improved by customising the models for the known dependencies of particular species. With a straightforward extension of the development model to cover the complete life cycle (with egg laying and density dependence), it is possible to incorporate field observations of eggs or adult mosquitoes, and develop an environment-driven population dynamics model.

## Conclusions

There is an urgent need to unravel the intricate physiological links between mosquitoes and their environment to quantify the impact of climate warming and control the future spread of disease. Here, we described an inverse modelling approach combining a pseudo-stage-structured population dynamics model and a semi-field design of life table experiments. The model allows for variability in development rate during the process, and is suitable for representing insect life cycles, subjected to highly variable environmental conditions. The combination can be used to accurately characterise a wide range of external drivers without the need to collect large amounts of data. Consequently, our approach complements the analytical and experimental methods needed for developing predictive large-scale climate-driven models for many insect species, such as those important for disease transmission, species conservation, and forensic investigation.

## Supplementary Information

Supplementary Information.

## Data Availability

The data generated and the code for analysis are available in the GitHub repository https://doi.org/10.5281/zenodo.6645355.
