# Supplementary material for: A dynamically structured matrix population model for insect life histories observed under variable environmental conditions
Source: Sci Rep. 2022 Jul 8;12:11587. doi: 10.1038/s41598-022-15806-2 (PMC9270365; doi:10.1038/s41598-022-15806-2)
Supplement: Supplementary file 1 — Supplementary Information. [file 41598_2022_15806_MOESM1_ESM.pdf]

Supplementary material: A dynamically structured matrix population model for insect life histories observed under variable environmental conditions

1 Development time distributions

The fixed scheme corresponds to the canonical degree-day development process[45]. Stage completion occurs after the accumulation of a precise number of degree-days, and the rate of accumulation is a function of environmental drivers, such as temperature. The fixed development scheme is a deterministic process with a target development time of k steps, where the rate of accumulation is $1/k$ per step.

The Pascal scheme, on the other hand, is designed to yield a Pascal-distributed development time. This is a special case of the negative binomial distribution, which can be thought of as the distribution of the number of failures (*i.e.* time steps) before k successes accumulate in a series of identical Bernoulli trials with probability of success θ. To indicate time, at least one failure event is required to be the final outcome in the series. Accordingly, q increases by i/k per step, where i is the number of successes at each step.

To simulate this process, we write the probability of obtaining x successes before 1 failure for each time step as

$$\Pr(i = x) = (1-\theta)\theta^x \tag{15}$$

and the cumulative density function as

$$\Pr(i \leq x) = F(x,\theta) = 1-\theta^{x+1}. \tag{16}$$

The Erlang scheme is the final scheme, implemented to yield Erlang-distributed development times. This is a special case of the gamma distribution, where development time corresponds to the waiting time for the kth renewal event in a Poisson process with exponentially distributed interarrival times. Consequently, at each time step, the number of events, i, is given by a Poisson-distributed random number (with rate $1/\theta$) and q is incremented by i/k. The cumulative density function for event arrival can be written as

$$\Pr(i \leq x) = F(x,\theta) = \frac{\Gamma(x+1, 1/\theta)}{x!},$$

where $\Gamma(x,\theta)$ is the upper incomplete gamma function.

To demonstrate the three schemes, we conjectured a small population of 10 individuals, expected to complete a life stage in 20 steps. We incorporated intrinsic stochastic variability in the form of a Pascal and an Erlang distribution with mean $\mu = 20$ and standard deviation $\sigma = 10$ steps, *i.e.* 20 ± 10, corresponding to $\theta = 0.2$ and $k = 5$ for Pascal and $\theta = 5.0$ and $k = 4$ for Erlang. To obtain the three development schemes and expose the impact of intrinsic stochasticity, we simulated the process 1000 times under the assumption of no birth, death, or other biological processes. As seen in the figures below, we demonstrate that Algorithm 1 accurately simulates fixed, Pascal-distributed, and Erlang-distributed development durations.

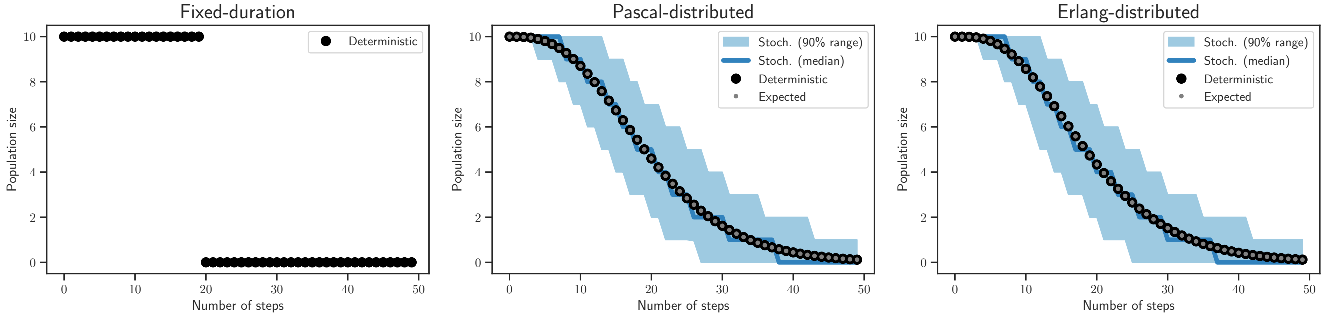

2 The dynamics of the population structure

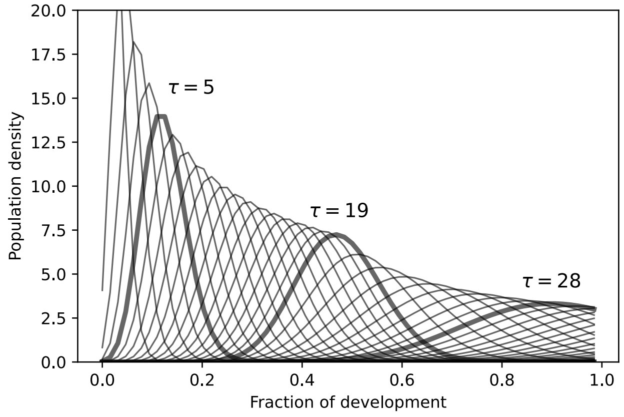

Figure S1. Progress of the development indicator. The frequency distribution of the development indicator, q, is shown for each time step, τ.

3 Accuracy of the pseudo-stage approximation

The advantage of q over i as the development indicator is that q provides a standard measure and allows for both k and θ to change. However, being a real-valued indicator between 0 and 1, q potentially, with a highly variable k, leads to an infinite number of pseudo-stages. In the stochastic setting, individuals are randomly assigned to a specific pseudo-stage, so the number of non-empty pseudo-stages never exceeds the number of individuals in a population. In contrast, a population can be divided indefinitely in the deterministic setting to represent the expected fraction in each pseudo-stage, and thus, the number of non-empty pseudo-stages can grow without bound. Under such circumstances, an approximation may be imposed by limiting the precision of q, which effectively limits the maximum number of pseudo-stages and groups individuals of almost identical progress together.

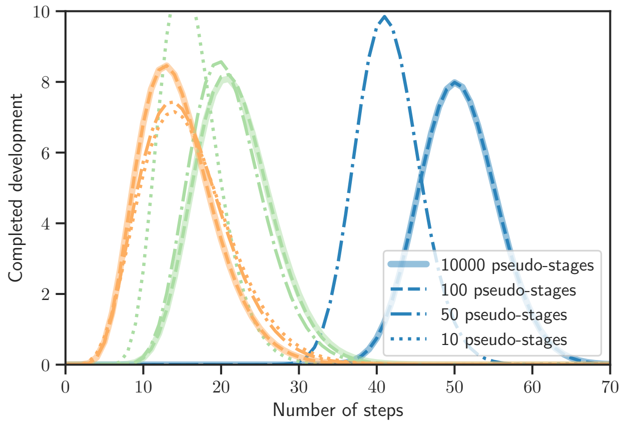

Figure S2. Limiting the number of pseudo-stages. The medians of development trajectories shown in Figure 2(b) when the number of pseudo-stages are limited by tuning the precision of the development indicator q. The significant decimal point is shown in parentheses.

4 Shifting the unit of time

In the context of matrix population modelling, a time step is a unit based on which the survival and development processes are defined. Time unit for daily survival probability, λ, can be scaled according to the relation,

$$\lambda' = \lambda^{1/\tau}, \tag{17}$$

which imposes a scale down by a factor of τ. On the other hand, the mean and standard deviation of a cumulative development process can be scaled according to

$$\mu' = \mu\,\tau \quad \text{and} \quad \sigma' = \sigma\,\tau. \tag{18}$$

Time scaling results in a change in k with the fixed scheme, in θ with the Erlang, and in both with the Pascal scheme. We note that a finer time scale may result in a deviation from the target probability distribution when using the Pascal scheme due to the change in the shape parameter, k. Therefore, extra care must be taken when defining a cumulative process with the Pascal scheme.

5 The variable-rate development model in R

```
  #
  # Copyright (C) 2022  Kamil Erguler
  #
  #   This program is free software: you can redistribute it and/or modify
  #   it under the terms of the GNU General Public License as published by
  #   the Free Software Foundation, either version 3 of the License, or
  #   any later version.
  #
  #   This program is distributed in the hope that it will be useful,
  #   but WITHOUT ANY WARRANTY; without even the implied warranty of
  #   MERCHANTABILITY or FITNESS FOR A PARTICULAR PURPOSE.  See the
  #   GNU General Public License for more details (<https://www.gnu.org/licenses/>).
  #
  # This code has been tested on GNU/Linux and OS X systems.
  #

library(stagePop)
solver.options=list(DDEsolver='PBS',tol=1e-8,hbsize=1e8,dt=0.01)

immigration <- function(mn,std,time) {
    theta <- std * std / mn
    k <- mn / theta
    return(dgamma(time,k,scale=theta))
}

solveDDE <- function(R1, R2) {
    init <- 100
    Tinit <- -40
    #
    ccFunctions <- list(
        deathFunc=function(stage,x,time,species,strain){return(0)},
        reproFunc=function(x,time,species,strain){return(0)},
        emigrationFunc=function(stage,x,time,species,strain){return(0)},
        develFunc=function(stage,x,time,species,strain){ # CONTINUOUS
            if (time > Tinit) {
                if (time < 20) {
                    if (stage == 1) {return(1/R1)} else {return(1e-13)}
                } else {
                    if (stage == 1) {return(1/R2)} else {return(1e-13)}
                }
            }
```

```
            return(1/R1)
        },
        durationFunc=function(stage,x,time,species,strain){ # INITIAL
            if (time == Tinit) {
                if (stage == 1) {return(R1)} else {return(1e13)}
            }
        },
        immigrationFunc=function(stage,x,time,species,strain){
            if (stage==1 && time<=0) {return(v <- immigration(40,5,time-Tinit+20))}
            return(0)
        }
    )
    #
    out <- popModel(
        numSpecies=1,
        numStages=2,
        timeDependLoss=FALSE,
        timeDependDuration=TRUE,
        ICs=list(matrix(0,nrow=2,ncol=1)),
        timeVec = seq(Tinit,50,0.01),
        solverOptions=solver.options,
        rateFunctions=ccFunctions,
        stageNames=list(c('juveniles','adults')),
        speciesNames=c('Culex'),
        saveFig = FALSE,
        plotFigs = FALSE
    )
    #
    return(data.frame("time"=out[,1],
                      "size"=out[,2]))
}

outS <- solveDDE(40,40) # 20+-5
outM <- solveDDE(60,40)
outL <- solveDDE(60,60) # 40+-5
```

6 The population dynamics model in C

```
 /*
  * Copyright (C) 2022  Kamil Erguler
  *
  *   This program is free software: you can redistribute it and/or modify
  *   it under the terms of the GNU General Public License as published by
  *   the Free Software Foundation, either version 3 of the License, or
  *   any later version.
  *
  *   This program is distributed in the hope that it will be useful,
  *   but WITHOUT ANY WARRANTY; without even the implied warranty of
  *   MERCHANTABILITY or FITNESS FOR A PARTICULAR PURPOSE.  See the
  *   GNU General Public License for more details (<https://www.gnu.org/licenses/>).
  *
  * This code has been tested on GNU/Linux and OS X systems.
  * It requires the sPop2 package
  *   https://doi.org/10.5281/zenodo.5788377
  * and the GNU Scientific Library (GSL)
  *   https://www.gnu.org/software/gsl/
  */

#include <math.h>
#include "spop2/spop2.h"
```

```
// Equations 1 and 2 (see main text)
#define funmort(T,Tm,a,c) (max(0.0,min(1.0, exp(a)*(pow((T)-(Tm),4.0)) + (c))))
#define briere1C(T,T0,T1,a) ( (T) <= (T0) ? 1e13 : ( (T) >= (T1) ? 1e13 : min(1e13,
    max(1.0, 1.0/(exp(a)*(T)*((T)-(T0))*sqrt((T1)-(T))) ) ) ))
#define briere1(T,T0,T1,a) (briere1C(273.15+(T),273.15+(T0),273.15+(T1),(a)))
// Equation 3 (see main text)
#define funphoto(P,PT,PS,S) (1.0 + ((PS)/(1.0 + exp((S)*((P)-(PT))))))

// Functional dependencies of mortality and development on temperature and photoperiod
void f_ph(double ph, double *p, double *m) {
  *m = funphoto(ph, p[24], p[25], p[26]);
}
void f_p1(double x, double ph, double *p, double *m) {
  *m = funmort(x, p[0], p[1], p[2]);
}
void f_p2(double x, double ph, double *p, double *m) {
  *m = funmort(x, p[3], p[4], p[5]);
}
void f_p3(double x, double ph, double *p, double *m) {
  *m = funmort(x, p[6], p[7], p[8]);
}
void f_p4(double x, double ph, double *p, double *m) {
  *m = funmort(x, p[9], p[10], p[11]);
}
void f_d1ms(double x, double ph, double *p, double *m, double *s) {
  *m = briere1(x, p[12], p[13], p[14]);
  *s = p[15] * (*m);
}
void f_d2ms(double x, double ph, double *p, double *m, double *s) {
  // Generic model (see main text)
  *m = briere1(x, p[16], p[17], p[18]);
  if ((p[24] > 0) & (p[25] > 0) & (p[26] > 0)) {
    // Extended model (see main text)
    double scl;
    f_ph(ph,p,&scl);
    *m *= scl;
  }
  *s = p[19] * (*m);
}
void f_d3ms(double x, double ph, double *p, double *m, double *s) {
  *m = briere1(x, p[20], p[21], p[22]);
  *s = p[23] * (*m);
}

// Print out the current state of the population
void print_out(int tm, spop2 *egg, spop2 *larva, spop2 *pupa, spop *adult, double *ret) {
  ret[tm*8+0] = (*egg)->size.d; // Total number of eggs
  ret[tm*8+1] = (*larva)->size.d; // Total number of larvae
  ret[tm*8+2] = (*pupa)->size.d; // Total number of pupae
  ret[tm*8+3] = (*adult)->size.d; // Total number of adults
  ret[tm*8+4] = 0.0; // Newly produced eggs
  ret[tm*8+5] = (*egg)->developed.d; // Newly produced larvae
  ret[tm*8+6] = (*larva)->developed.d; // Newly produced pupae
  ret[tm*8+7] = (*pupa)->developed.d; // Newly produced adults
}
// Print out zeros as the current state
void print_zero(int tm, double *ret) {
  ret[tm*8+0] = 0.0;
  ret[tm*8+1] = 0.0;
  ret[tm*8+2] = 0.0;
```

```
  ret[tm*8+3] = 0.0;
  ret[tm*8+4] = 0.0;
  ret[tm*8+5] = 0.0;
  ret[tm*8+6] = 0.0;
  ret[tm*8+7] = 0.0;
}

void sim(int tf, double *temp, double *photo, double *pr, double *y0, double thr, double
    *ret) {
  // Set the maximum number of pseudo states to 100
  set_APPROX(1e-2);

  double p1=0.0, p2=0.0, p3=0.0, p4=0.0, d1m=0.0, d1s=0.0, d2m=0.0, d2s=0.0, d3m=0.0,
      d3s=0.0;

  // Erlang-distributed egg, larva, and pupa development (spop2 represents cumulative
      development)
  spop2 egg   = spop2_init(0, MODE_ACCP_ERLANG);
  spop2 larva = spop2_init(0, MODE_ACCP_ERLANG);
  spop2 pupa  = spop2_init(0, MODE_ACCP_ERLANG);
  // Daily mortality for adults (spop represents geometrically-distributed adult lifetime)
  spop adult  = spop_init(0, MODE_GAMMA_HASH);

  // Initialisations
  int tm = 0;
  if (y0[0]) spop2_add(egg,   0, y0[0]);
  if (y0[1]) spop2_add(larva, 0, y0[1]);
  if (y0[2]) spop2_add(pupa,  0, y0[2]);
  if (y0[3]) spop_add(adult,  0, 0, 0, y0[3]);
  print_out(tm, &egg, &larva, &pupa, &adult, ret);

  // Perform life processes for tf-1 steps
  for (tm=1; tm<tf; tm++) {
    // Calculate survival rates
    f_p1(temp[tm-1],photo[tm-1],pr,&p1);
    f_p2(temp[tm-1],photo[tm-1],pr,&p2);
    f_p3(temp[tm-1],photo[tm-1],pr,&p3);
    f_p4(temp[tm-1],photo[tm-1],pr,&p4);

    // Calculate development rates
    f_d1ms(temp[tm-1],photo[tm-1],pr,&d1m,&d1s);
    f_d2ms(temp[tm-1],photo[tm-1],pr,&d2m,&d2s);
    f_d3ms(temp[tm-1],photo[tm-1],pr,&d3m,&d3s);

    // Impose survival and development
    spop2_iterate(egg,   d1m, d1s, p1, 0);
    spop2_iterate(larva, d2m, d2s, p2, 0);
    spop2_iterate(pupa,  d3m, d3s, p3, 0);
    spop_iterate(adult,    0, 0, 0, 0,  p4, 0, 0, 0,  0);

    // Perform state changes
    spop_add(adult,  0, 0, 0, pupa->developed.d);
    spop2_add(pupa,  0, larva->developed.d);
    spop2_add(larva, 0, egg->developed.d);

    // Print out the current state; or, if almost no immature stage left, print out zeros
    print_out(tm, &egg, &larva, &pupa, &adult, ret);
    if (thr > 0 && egg->size.d < thr && larva->size.d < thr && pupa->size.d < thr) {
      for (tm++; tm<tf; tm++)
      print_zero(tm, ret);
```

```
      break;
    }
  }

  // Destroy the memory allocated to the development stages
  spop2_destroy(&egg);
  spop2_destroy(&larva);
  spop2_destroy(&pupa);
  spop_destroy(&adult);
}
```

7 Life histories under constant conditions

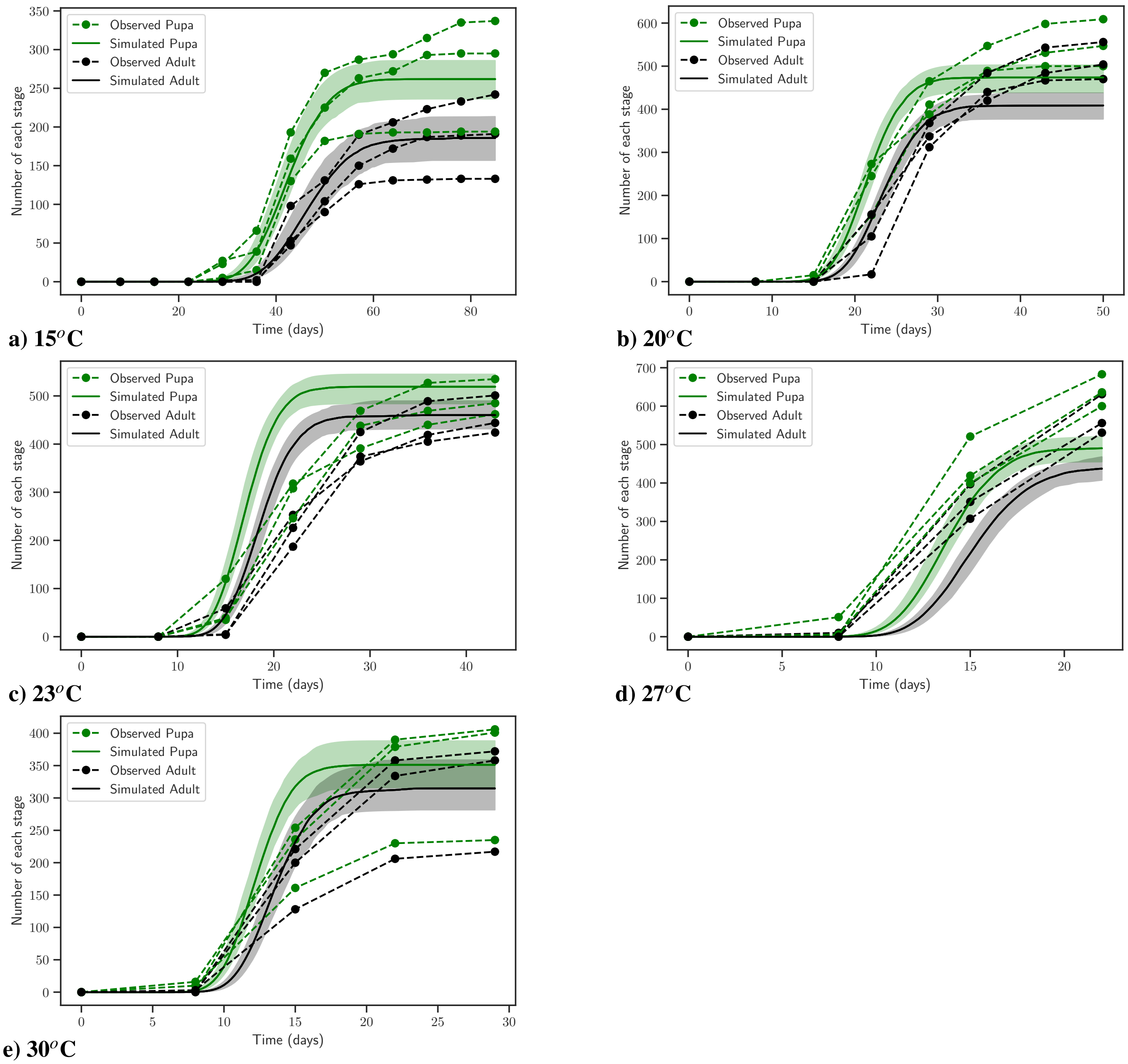

Figure S3. Observed and simulated life histories of *Cx. quinquefasciatus* under constant conditions. Solid lines represent the median and shaded areas represent the 90% range of pupa (green) and adult (black) production.

8 Life histories under variable conditions

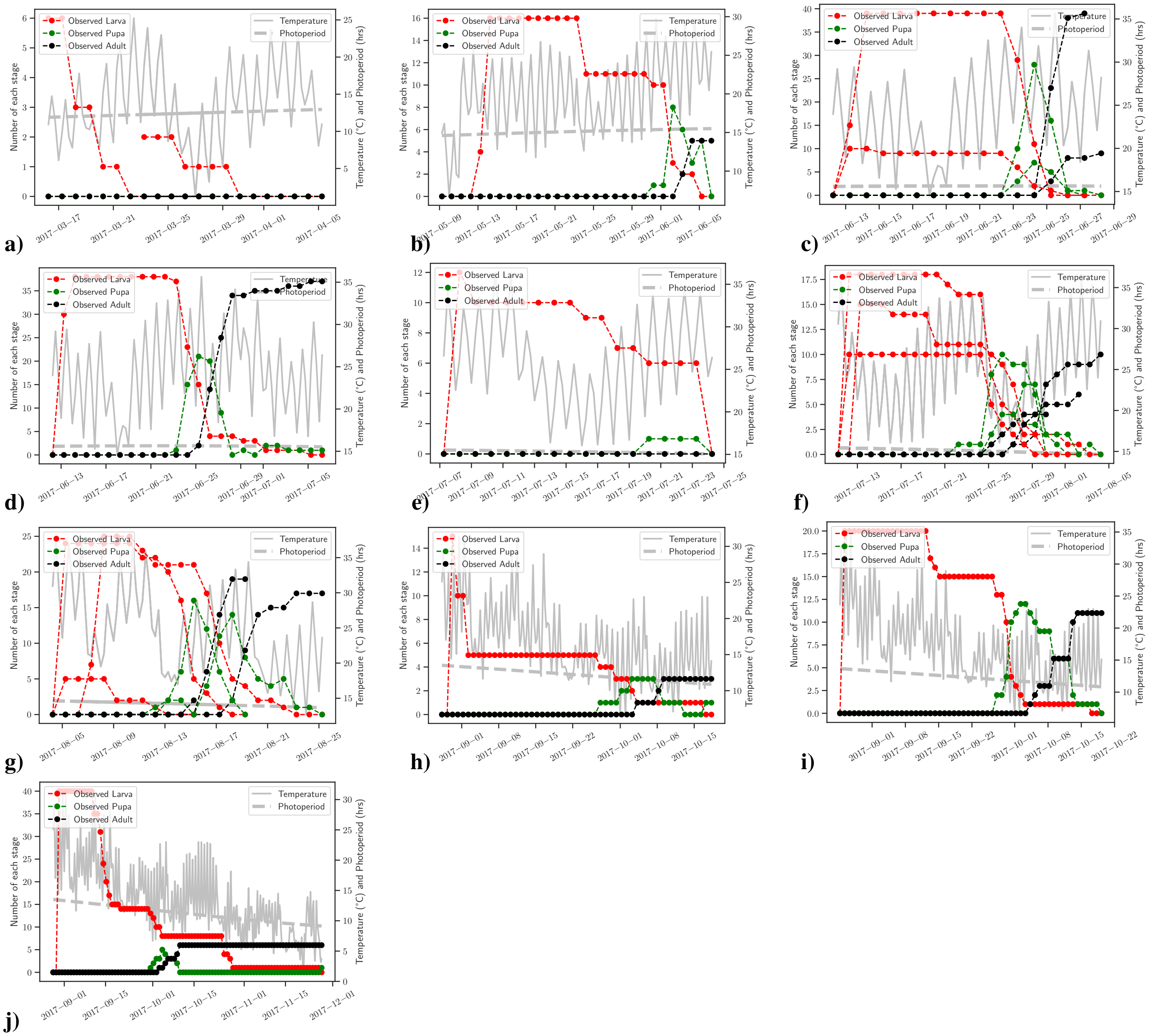

Figure S4. **Observed life histories of *Cx. pipiens* under variable conditions.**

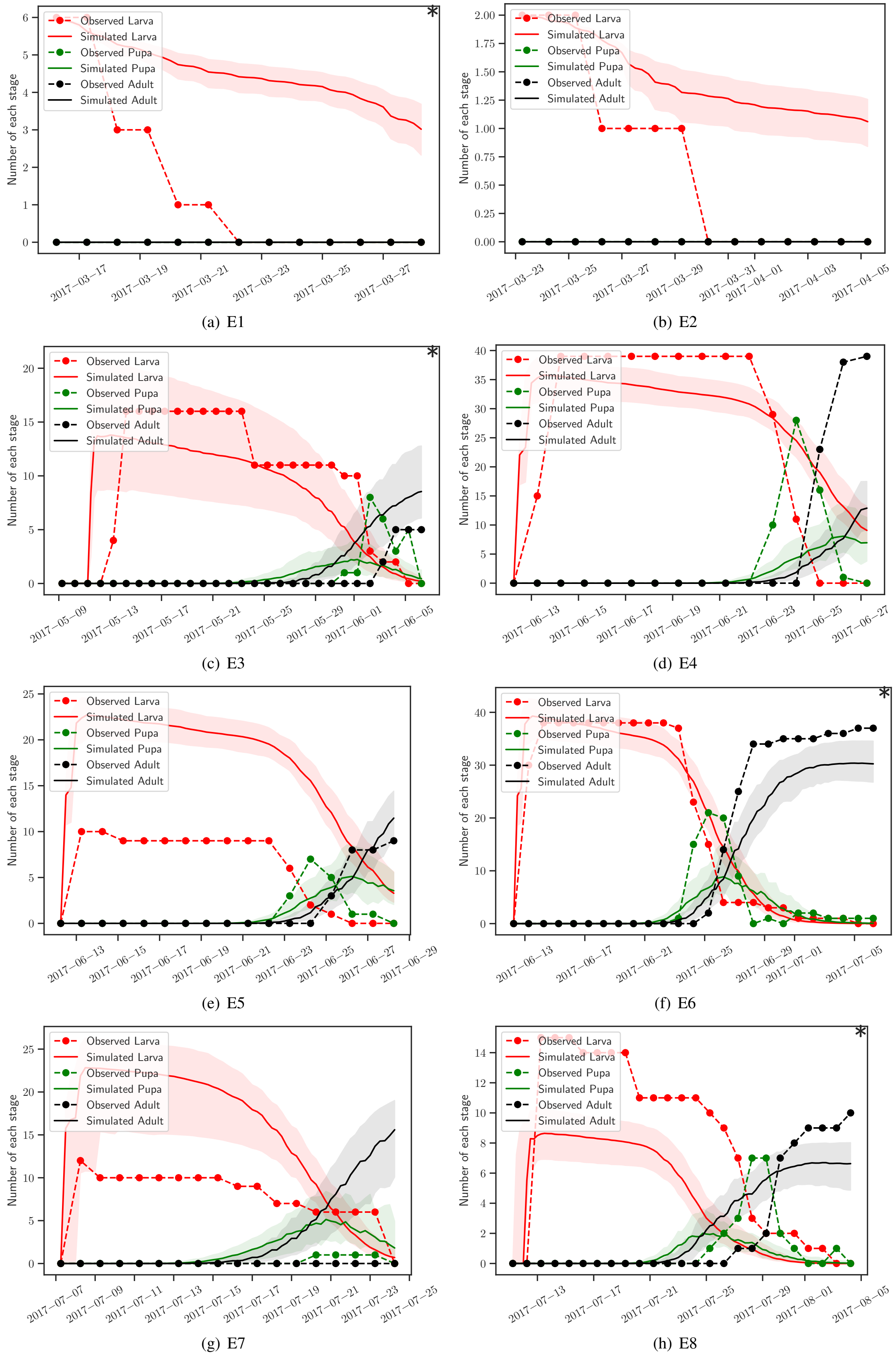

Figure S5. Observed and simulated life histories of *Cx. pipiens*. Solid lines represent the median and shaded areas represent the 90% range. (*) indicates data included in calibration.

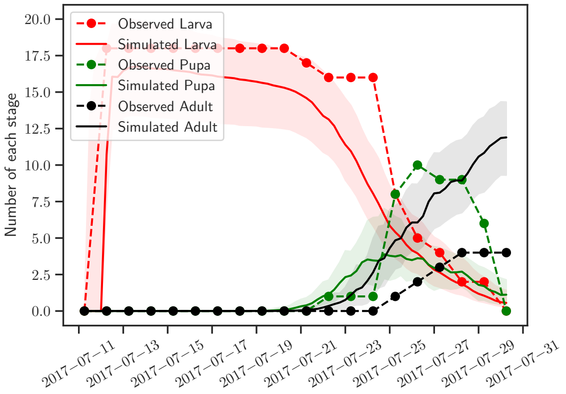

(a) E9

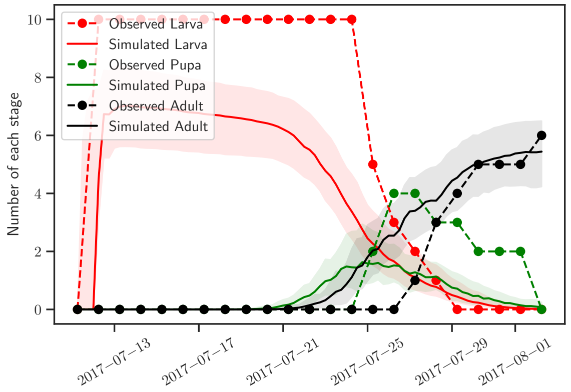

(b) E10

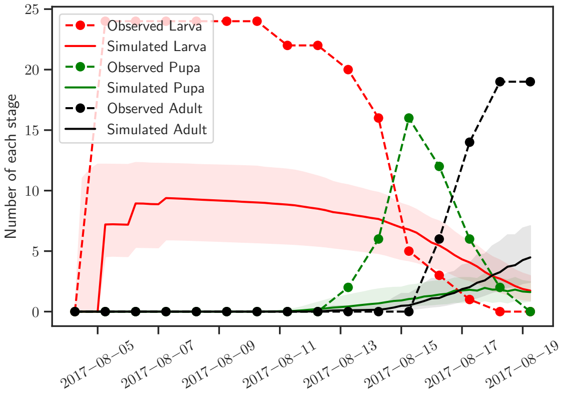

(c) E11

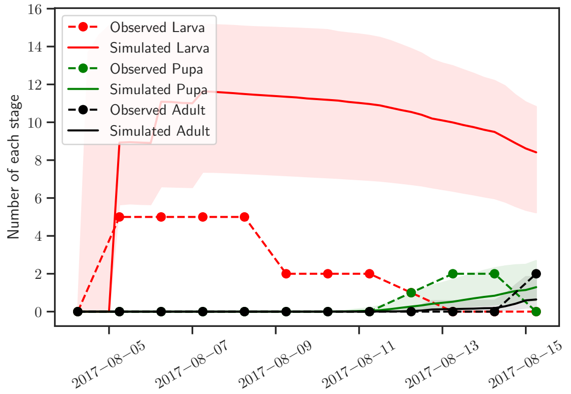

(d) E12

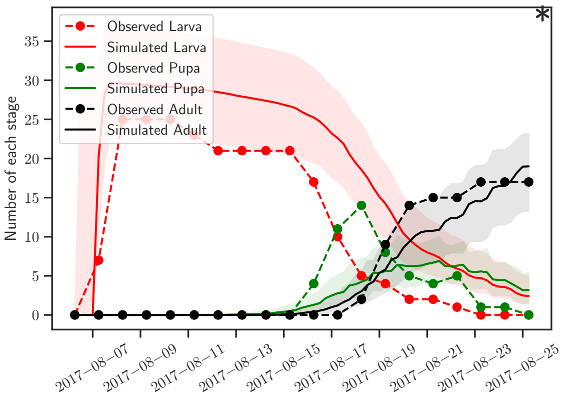

(e) E13

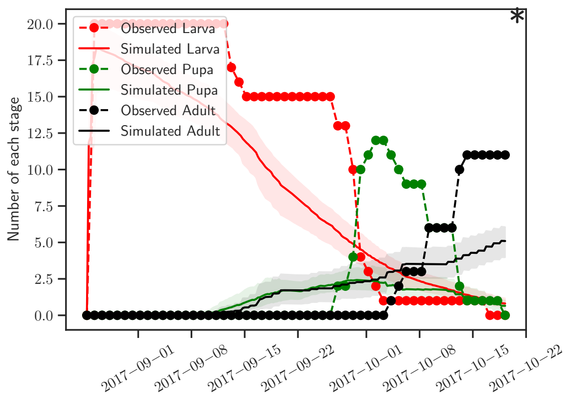

(f) E14

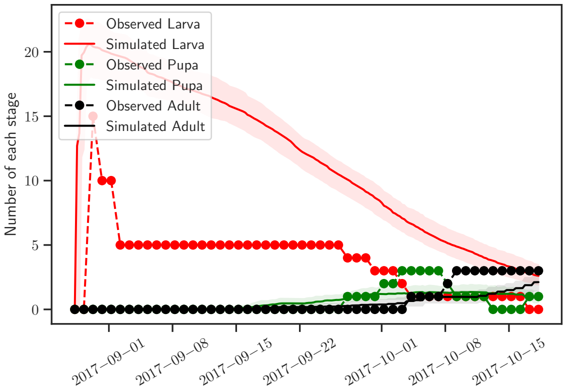

(g) E15

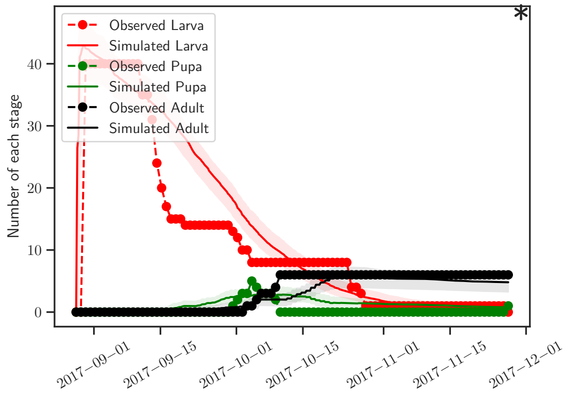

(h) E16

Figure S6. Observed and simulated life histories of *Cx. pipiens*. Solid lines represent the median and shaded areas represent the 90% range. (*) indicates data included in calibration.

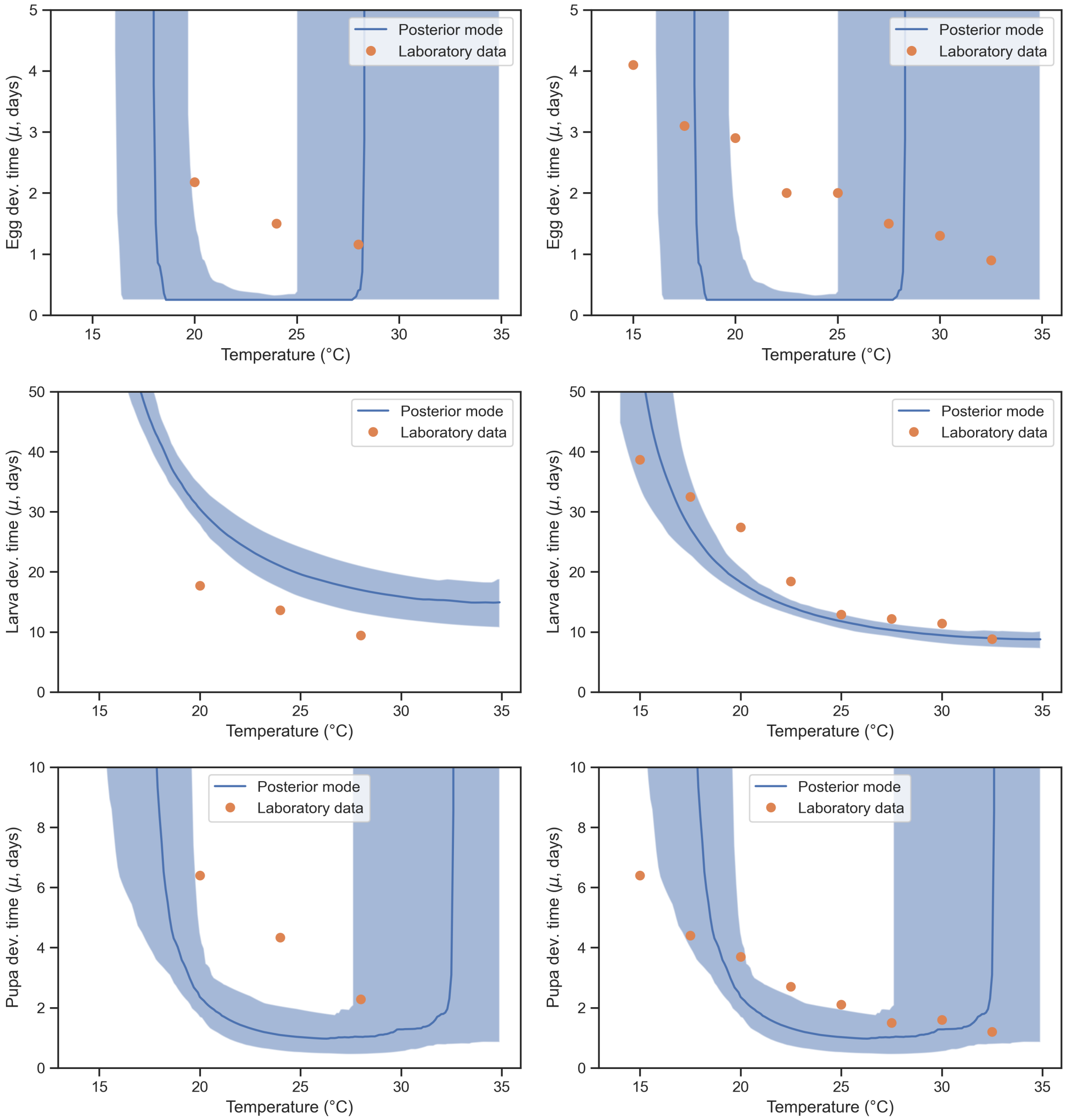

Figure S7. Observed and inferred environmental dependency of *Cx. pipiens* development and mortality. The left panel presents comparison with Kiarie-Makara *et al.* (2015)[84] (13:11 h L:D) and the right panel with Spanoudis *et al.* (2019)[62] (16:8 h L:D). Solid lines represent the median and shaded areas represent the 90% range.

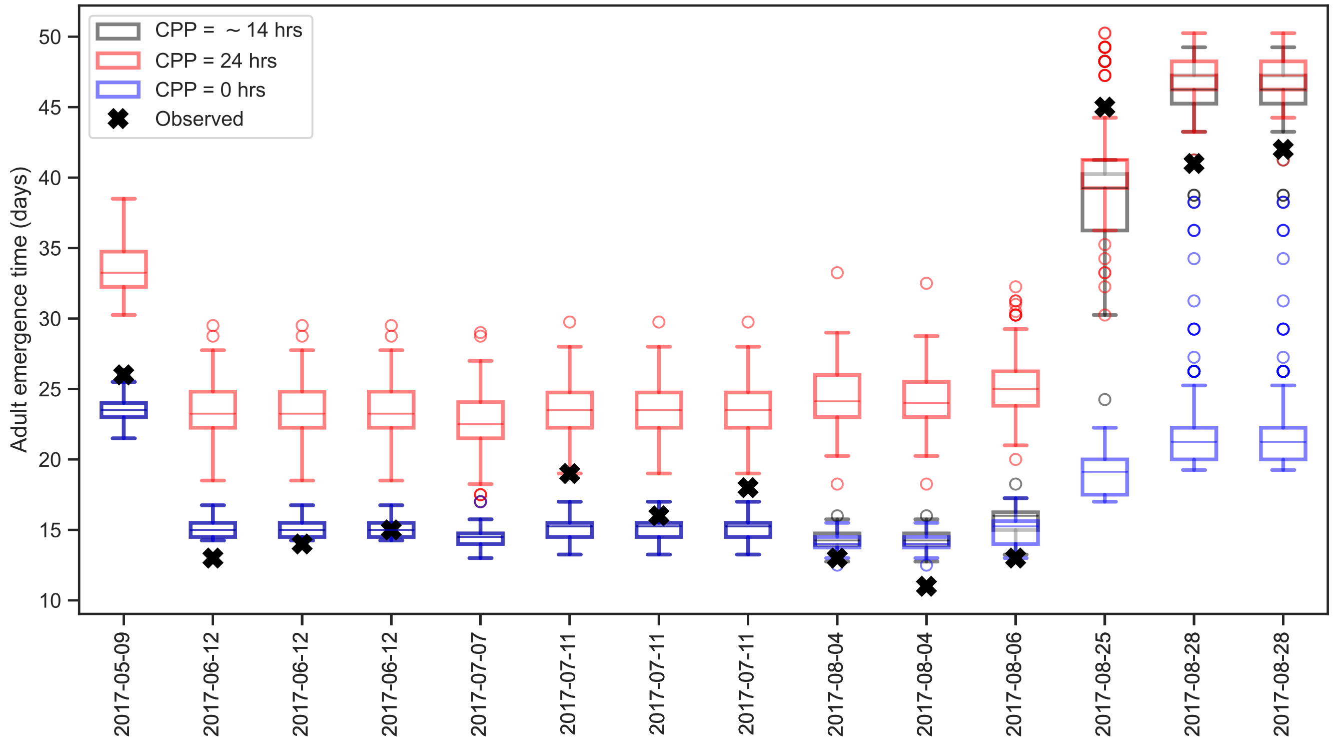

Figure S8. Development with alternative critical photoperiods (CPPs) for *Cx. pipiens*.

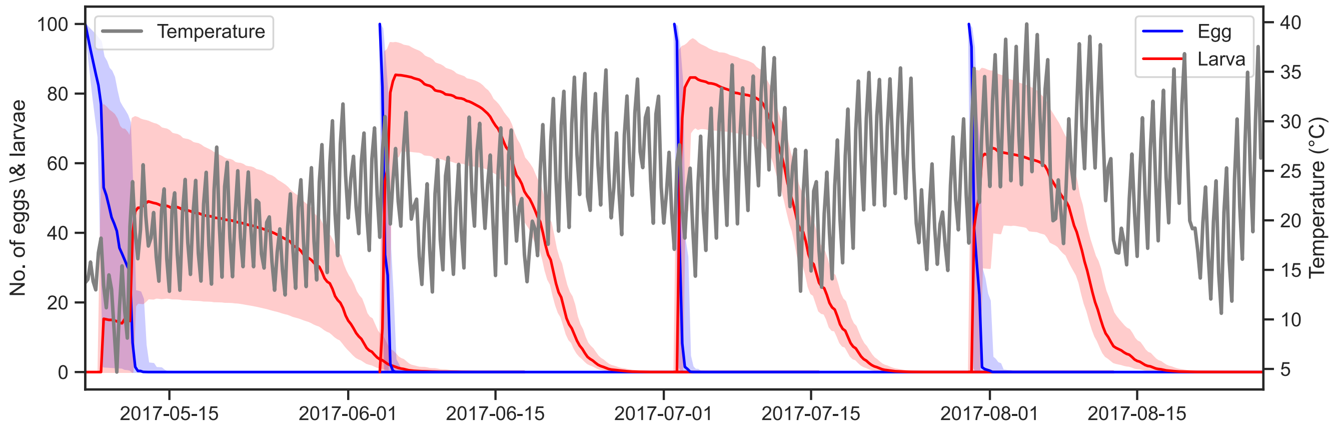

Figure S9. Early development of *Cx. pipiens* initiated at several weeks in Petrovaradin, Serbia, in 2017. Solid lines represent the median and shaded areas represent the 90% range.
